# “A” stands for airway – Which factors guide the need for on-scene airway management in facial fracture patients?

**DOI:** 10.1186/s12873-022-00669-7

**Published:** 2022-06-15

**Authors:** Tero Puolakkainen, Miika Toivari, Tuukka Puolakka, Johanna Snäll

**Affiliations:** 1 Department of Oral and Maxillofacial Diseases, University of Helsinki and Helsinki University Hospital, P.O. Box, 100, FI-00029 HUS Helsinki, Finland; 2 Department of Emergency Medicine and Services, University of Helsinki and Helsinki University Hospital, Helsinki, Finland; 3 Department of Anaesthesiology and Intensive Care Medicine, University of Helsinki and Helsinki University Hospital, Helsinki, Finland

**Keywords:** Intubation, Airway, Facial Fracture, Trauma, Traumatic Brain injury

## Abstract

**Background:**

Numerous guidelines highlight the need for early airway management in facial trauma patients since specific fracture patterns may induce airway obstruction. However, the incidence of these hallmark injuries, including flail mandibles and posterior displacement of the maxilla, is contentious. We aim to evaluate specific trauma-related variables in facial fracture patients, which affect the need for on-scene versus in-hospital airway management.

**Methods:**

This retrospective cohort study included all patients with any type of facial fracture, who required early airway management on-scene or in-hospital. The primary outcome variable was the site of airway management (on-scene versus hospital) and the main predictor variable was the presence of a traumatic brain injury (TBI). The association of fracture type, mechanism, and method for early airway management are also reported. Altogether 171 patients fulfilled the inclusion criteria.

**Results:**

Of the 171 patients included in the analysis, 100 (58.5) had combined midfacial fractures or combination fractures of facial thirds. Altogether 118 patients (69.0%) required airway management on-scene and for the remaining 53 patients (31.0%) airway was secured in-hospital. A total of 168 (98.2%) underwent endotracheal intubation, whereas three patients (1.8%) received surgical airway management. TBIs occurred in 138 patients (80.7%), but presence of TBI did not affect the site of airway management. Younger age, Glasgow Coma Scale-score of eight or less, and oro-naso-pharyngeal haemorrhage predicted airway management on-scene, whereas patients who had fallen at ground level and in patients with facial fractures but no associated injuries, the airway was significantly more often managed in-hospital.

**Conclusions:**

Proper preparedness for airway management in facial fracture patients is crucial both on-scene and in-hospital. Facial fracture patients need proper evaluation of airway management even when TBI is not present.

## Background

Numerous guidelines highlight the significance of early airway assessment and management in trauma patients [1, 2]. A compromised airway leads to impaired respiratory function with devastating sequelae, potentially directly affecting the morbidity and mortality of these patients. This emphasizes the importance of airway management already in a prehospital setting [3, 4].

Hallmark injury profiles in facial fracture patients requiring primary endotracheal intubation include posterior displacement of the fractured maxilla or flail mandibles, which are complicated by intractable fracture site haemorrhage [5, 6]. Critical airway obstruction typically occurs in panfacial fractures, however, isolated mandibular fractures with inward displacement of the genial tubercle and floor of the mouth may also contribute to this [7, 8].

Patients with facial trauma comprise an especially demanding patient population in terms of airway management. The endotracheal intubation of trauma patients is often challenged by haemorrhage, gastric content, soft tissue swelling, and foreign material in the mouth and upper airway tract [9]. In addition, patients sustaining injuries to the facial region are at particular risk of airway obstruction and the feared “can not intubate, can not ventilate”-scenario [10].

Another essential perspective is the presence of cranial injuries and injuries to the central nervous system in particular such as traumatic brain injuries (TBI). In fact, low Glasgow Coma Scale (GCS), a score used to primarily evaluate TBI presence, –scores (8 or lower) are independent criteria indicating the need for airway management in craniofacial trauma patients [1]. On the other hand, facial facture patients are at a high risk of sustaining concomitant TBI but their role in predicting early airway management is not well known [11].

Additionally, the presence of cervical spine injuries and blunt cerebrovascular injuries also needs to be considered [12, 13]. Therefore, repeated assessments of the patient’s neurological responses are warranted, and preparedness for rapid airway management when necessary.

The purpose of this study was to describe the clinically relevant variables affecting airway management of patients with different types of facial fractures, and factors associated with on-scene and in-hospital airway management. Our hypothesis was that patient sustaining facial fractures are at a high risk of requiring on-scene airway management regardless of TBI presence.

## Methods

### Study design and inclusion and exclusion criteria

The records of all patients with any type of facial fracture admitted to two tertiary trauma centres (Trauma Unit of Helsinki University Hospital or Children’s Hospital, Helsinki, Finland, catchment area of around two million inhabitants) between 1 January 2013 and 31 December 2018 were evaluated retrospectively. Patients records were searched for by corresponding ICD-codes from the local electronic database (Diagnosis code S02: fracture of skull and facial bones). Each patient record was screened for the study variables described below including specific facial fracture type, site of airway management and presence of TBI.

Patients who underwent primary airway management (i.e. endotracheal intubation, cricothyroidotomy or tracheostomy) and had any radiologically confirmed facial fracture were included in the study. Patients who sustained their injuries in other countries were excluded from the study as their initial management had already been provided elsewhere. Early airway management was defined as managing airway on-scene or in-hospital due to the threat of airway obstruction after trauma and not electively prior to operative treatment

The emergency medical services (EMS) consists of basic and advanced life support ambulance units. In addition, one physician-staffed mobile intensive care unit (MICU) and one helicopter-based unit are involved. All prehospital patients are approached according to prehospital guidelines and patient evaluation based on the ABCDE-algorithm is implemented. All prehospital patients were intubated by critical care physicians with training in anaesthesiology. In-hospital airway management was performed by anaesthesiologists, traumatologists, or oral and maxillofacial surgeons of the trauma team. The selection of proper airway management method and equipment (i.e. standard or video laryngoscopy) was based on the physician’s clinical evaluation. Both MICUs were equipped with video laryngoscopes.

### Study variables

The main outcome variable was site of airway management (on-scene or in-hospital).

The main predictor variable was presence of TBI which was defined as any radiographically confirmed intracranial injury.

The secondary predictor variable was the patient’s injury profile. Based on initial assessment, patients were grouped according to their injury profiles into patients with “multiple injuries” (patients with any associated injury in addition to facial fracture), patients with “only associated head injuries” (only associated cranial fracture and/or TBI in addition to facial fracture), and patients with “isolated facial fractures”. Associated injuries were defined as injury of extremities (including fractures and joint dislocations of the upper and lower extremities), torso injuries (thoracic, abdominal and pelvic injuries) and neck injuries (cervical spine injuries and blunt cerebrovascular injuries), any type of cranial fracture and TBI.

Additional predictor variables were the presence of oro-naso-pharyngeal haemorrhage (ONP-haemorrhage) and facial fracture type. Facial fractures were classified as mandibular, nasal, combined midfacial, combination of facial thirds, upper facial third, and unilateral zygomatic-maxillary-orbital (ZMO-) fractures. ZMO -fractures included unilateral zygomatic, maxillary and orbital fractures as well as combinations thereof.

Additionally, methods for primary airway management (endotracheal intubation and surgical airway consisting of cricothyroidotomy and tracheostomy) and primary intubation attempts are reported.

Explanatory variables were age, sex, injury mechanism, and primary GCS -score (grouped as 9 or more, 3-8, and unknown).

#### Statistical analysis

Descriptive statistics are presented as absolute values and percentages. Continuous variables that were not normally distributed were analyzed using Mann-Whitney U test. Pearson’s Chi Square test or Fisher’s Exact test were used to assess the significance of differences in categorical variables. Statistical significance was set at 0.05. Statistical tests were performed using SPSS version 25.0 (IBM Corp., NY, USA).

#### Ethical considerations

The study was approved by the Internal Review Board of the Head and Neck Center, Helsinki University Hospital, Helsinki, Finland (HUS/356/2017 and HUS/54/2019).

## Results

Out of 2919 facial fracture patients, 171 (5.9%) received primary airway management and were included in the final analysis Table 1. Males were over-represented in this study population at 78.9%. The median age for all patients was 40.0 years, and 16 patients (9.4%) were under the age of 18 years.

**Table 1:** Descriptive statistics of 171 patients with facial fractures needing airway management after initial trauma

Descriptive variables	N	%
**Total no. of patients**	171	100
**Sex**		
Male	135	78.9
Female	36	21.1
**Age (years)**		
Median (IQR)	40.0 (31.2)	
Range	3.6 – 88.4	
<18 years	16	9.4
≥18 years	155	90.6
**Injury mechanism**		
Ground-level fall	15	8.8
Fall from height	44	25.7
Fall from stairs	15	8.8
Motor vehicle accident	53	31.0
Bicycle accident	13	7.6
Assault	14	8.2
Other / Unknown	17	9.9
**Injury profile**		
Multiple injuries	112	65.5
Only associated head injuries	50	29.2
Isolated facial fracture	9	5.2
**Type of facial fracture**		
Combination of facial thirds	59	34.5
Combined midfacial	41	24.0
Unilateral zygomatic-maxillary-orbital	36	21.1
Isolated mandibular	13	7.6
Isolated upper third	13	7.6
Isolated nasal	9	5.3
**Presence of oro-naso-pharyngeal haemorrhage**		
No	91	53.2
Yes	80	46.8
**Site of airway management**		
Trauma scene	118	69.0
Emergency department/hospital	53	31.0
**Method of primary airway management**		
Surgical	3	1.8
Endotracheal intubation	168	98.2
**Attempts at endotracheal intubation**		
1	151	89.9
2	5	3.0
Not specified	12	7.1

The majority of patients had their airway managed on-scene (*n*=118/171, 69.0%). Of the 171 patients, three patients had their airway primarily managed surgically (1.8%) and 168 patients underwent oral or nasal endotracheal intubation (98.2%). Of these 168 patients, endotracheal intubation was successful on the first attempt in 151 (89.9%). Concerning the three patients requiring surgical airway management on-scene, two underwent cricothyroidectomies and one had the airway successfully managed by tracheostomy; all three had sustained severe panfacial fractures with a high risk of airway obstruction.

Motor vehicle accidents (31.0%) followed by falls from heights (25.7%) were the most common injury mechanisms. Based on injury profiles, 59 patients (34.4%) had isolated facial fractures or only associated head injuries. Fractures to different combinations of facial thirds (34.5%) and combined midfacial fractures (24.0%) were the most frequent facial fracture patterns. Presence of ONP -haemorrhage was detected in 80 patients (46.9%).

Patients with multiple injuries received on-scene airway management most frequently (77.7%, *p*=0.001) Table 2. Patients with only associated head injuries (i.e. cranial fracture and/or TBI in addition to facial fracture) required on-scene airway management in 54% of cases (*p*=0.006). However, as separately analyzed, TBI presence was non-significant when assessing the site of airway management in facial fracture patients (*p*=0.746).

**Table 2:** Comparison of patients based on the site where airway management took place.

Variable	Airway management at trauma scene			Airway management at hospital			***p***-value
	No. of patients	%	% of n	No. of patients	%	% of n	
Total	118	69.0		53	31.0		
**Age** (mean)	39.6			48.7			0.002
**Sex**							
Male	89	65.9	75.4	46	34.1	87.0	0.092
Female	29	80.6	24.6	7	19.4	13.0	
**Injury mechanism**							
Ground-level fall	6	40.0	5.1	9	60.0	17.0	0.018
Fall from height	30	68.2	25.4	14	31.8	26.4	0.891
Fall from stairs	11	73.3	9.3	4	26.7	7.5	1.000
Motor vehicle accident	42	79.2	35.6	11	20.8	20.8	0.052
Bicycle accident	9	69.2	7.6	4	30.8	7.5	1.000
Assault	7	50.0	5.9	7	50.0	13.2	0.134
Other / Unknown	13	76.5	11.0	4	23.5	7.5	0.483
**Primary Glasgow Coma Scale -value**							
≥ 9	27	41.5	22.9	38	58.5	71.7	<0.001
3-8	87	86.1	73.7	14	13.9	26.4	<0.001
Unknown	4	80.0	3.4	1	20.0	1.9	0.506
**Presence of TBI**							
No	22	18.6	66.7	11	20.8	33.3	0.746
Yes	96	81.4	69.6	42	79.2	30.4	
**Injury profile**							
Multiple injuries	87	77.7	73.7	25	22.3	47.2	0.001
Only associated head injuries	27	54.0	22.9	23	46.0	43.4	0.006
Isolated facial fracture	4	44.4	3.4	5	55.6	9.4	0.138
**Type of facial fracture**							
Combination of facial thirds	40	67.8	33.9	19	32.2	35.8	0.804
Combined midfacial	33	80.5	28.0	8	19.5	15.1	0.068
Unilateral zygomatic-maxillary-orbital	26	72.2	22.0	10	27.8	18.9	0.639
Isolated mandibular	8	61.5	6.8	5	38.5	9.4	0.544
Isolated upper third	6	46.2	5.1	7	53.8	13.2	0.114
Isolated nasal	5	55.6	4.2	4	44.4	7.5	0.461
**Oro-naso-pharyngeal haemorrhage**							
No	53	44.9	58.2	38	71.7	41.8	0.002
Yes	65	55.1	81.3	15	28.3	18.8	

Concerning patients with isolated facial fractures, four (44.4%) underwent airway management on-scene. Presence of ONP -haemorrhage was a strong indicator for on-scene airway management (81.3% of patients with ONP -haemorrhage, *p*=0.002). In addition, patients who required airway management on-scene were significantly younger than patients who had their airway managed at hospital (*p*=0.002). Almost 80% of patients injured in motor vehicle accidents had their airway managed on-scene (*p*=0.052), whereas 60.0% of patients who fell from ground level had their airway managed at hospital (*p*=0.018).

In all, 80.7% of facial fracture patients had TBI Table 3. Notably, 52 patients (37.7%) with TBI had an initial GCS value of 9 or more. In addition, 12 out of 13 patients (92.3%) who sustained their injuries in bicycle accidents were diagnosed with TBI. All patients with isolated fractures of the upper facial third sustained concomitant TBIs.

**Table 3:** Comparison of clinically relevant variables in patients with or without traumatic brain injuries

Variable	Facial fracture patients with TBI			Facial fracture patients without TBI			***p***-value
	No. of patients	%	% of n	No. of patients	%	% of n	
Total	138	80.7		33	19.3		
**Age** (mean)	42.7			41.2			0.595
**Sex**							
Male	113	83.7	81.9	22	16.3	66.7	0.054
Female	25	69.4	18.1	11	30.6	33.3	
**Mechanism**							
Ground-level fall	10	66.7	7.2	5	33.3	15.2	0.171
Fall from height	35	79.5	25.4	9	20.5	27.3	0.822
Fall from stairs	10	66.7	7.2	5	33.3	15.2	0.171
Motor vehicle accident	44	83.0	31.9	9	17.0	27.3	0.607
Bicycle accident	12	92.3	8.7	1	7.7	3.0	0.467
Assault	11	78.6	8.0	3	21.4	9.1	0.735
Other / Unknown	16	94.1	11.6	1	5.9	3.0	0.200
**Primary Glasgow Coma Scale -value**							
≥ 9	52	80.0	37.7	13	20.0	39.4	0.935
3-8	82	81.2	59.4	19	18.8	57.6	0.935
Unknown	4	80.0	2.9	1	20.0	3.0	1.000
**Type of facial fracture**							
Combination of facial thirds	49	83.1	35.5	10	16.9	30.3	0.572
Combined midfacial	34	82.9	24.6	7	17.1	21.2	0.679
Unilateral zygomatic-maxillary-orbital	28	77.8	20.3	8	22.2	24.2	0.617
Isolated mandibular	8	61.5	5.8	5	38.5	15.2	0.134
Isolated upper third	13	100.0	9.4	0	0.0	0.0	0.076
Isolated nasal	6	66.7	4.3	3	33.3	9.1	0.378
**Oro-naso-pharyngeal haemorrhage**							
No	72	52.2	79.1	19	57.6	21.9	0.698
Yes	66	47.8	82.5	14	42.4	17.5	

## Discussion

Patients with injuries to the head and neck region are particularly susceptible to having their airway compromised, and trauma surgeons are cognisant of how specific facial fractures may affect airway obstruction. However, there is a dearth of evidence on the parameters associated with facial fracture patients requiring primary airway management. In this study, we report novel data on specific trauma-related variables, including presence of TBI, in facial fracture patients and their relationship with the need for primary airway management. Our hypothesis was confirmed as presence of TBI did not significantly affect the need for on-scene airway management in patients with facial fractures.

A significant majority of the patients needing airway management at the trauma scene were diagnosed with fractures in different combinations of facial thirds (33.9%) and combined midfacial fractures (28.0%). Due to the anatomical proximity of specific branches and sub-branches of the external carotid artery, mid-facial fractures are among the most common injury types when excessive oropharyngeal bleeding occurs, and it has been suggested that life-threatening bleeding occurs in 1.2-4.5% of facial fracture patients [14, 15]. Dar and colleagues recently reported that 2.3% of maxillofacial trauma patients required transcatheter arterial embolization to treat life-threatening haemorrhage highlighting the significance of severe bleeding of the facial region [16]. In the present study, 55.1% of patients who required primary airway management presented with oropharyngeal haemorrhage. Depending on the severity of the injury, proper visualization of the anatomic landmarks may prove to be extremely difficult on-scene, prolonging the duration of airway obstruction. Despite this, almost 90% of endotracheal intubations in our study were successful on the first attempt. This could be due to many reasons, including the effective use of videolaryngoscopes in endotracheal intubation [17]. Only 1.8% (3 out of 171) of the included patients required on-scene surgical airway management. All three of these patients sustained severe panfacial fractures with imminent airway obstruction. Similar findings were reported by Aziz et al over a twenty-year period [18].

The majority of studies investigating airway management and facial fractures mostly involve the evaluation of specific methods for airway management in surgical procedures. However, it is paramount to emphasize that patients with facial fractures are at risk of airway obstruction immediately post-trauma. A well-cited report by Tung and colleagues described how only 1.7% of facial fracture patients had their airway compromised due to trauma. Of these patients, 47.1% had complex facial fractures and 17.6% isolated mandible fractures. However, it was not differentiated how many of these patients were the subject of polytrauma or other injuries. Another essential observation is that in the Tung et al. study, compared with our study setup, patients who required emergency endotracheal intubation were excluded from the study. Le et al. comprehensively evaluated the need for, and the type of airway management in a facial fracture population [19]. However, variables contributing to the need for primary airway management were not investigated. Therefore, the actual need for primary airway management in this patient population could be higher and requires further investigating. Moreover, this highlights the significance of the maxillofacial surgeon’s role in a multi-disciplinary trauma team.

In total, 69.0% of patients with facial fractures underwent primary airway management on-scene. Most of these patients were endotracheally intubated due to low GCS -scores or altered level of consciousness. However, it is important to acknowledge that in general, patients may also be intubated to protect against potential aspiration of gastric contents. Based on our results, patients with facial fractures who required airway management on-scene were significantly younger than patients who had their airway managed at hospital. Another curious finding was that most patients who sustained their facial injuries by falling at ground level required airway management at hospital and not on-scene.

Proper appraisal and diagnosis of TBI, especially in conscious patients in a primary trauma setting, can be extremely challenging since variations in patient behaviour and compliance, neurological status, and presence of other distracting injuries all need to be considered when evaluating the need for airway management [20]. GCS -scores and their applicability are widely accepted in primary trauma surveys and may even be an independent variable indicating airway management [1]. On the other hand, their use in specific trauma populations has been questioned [21]. Assessment of “altered mental status” may be particularly unreliable [22]. Based on our results, the presence of TBI in facial fracture patients did not affect whether airway management took place on-scene or in-hospital. Therefore, the presence of a facial fracture in patients with only associated head injuries is accentuated especially when assessing the need for airway management.

The main limitation of this study is its retrospective study design. Selection bias in cohorts is inevitable when assessing specific study aims from a limited population. On the other hand, there is a dearth of publications with similar study designs and our study provides an important overview of numerous variables and their relationship with primary airway management in facial fracture patients including the site of airway management. Additionally, most of the injury mechanisms are related to blunt-trauma and these findings cannot be compared with datasets on, for instance, severe gunshot wound injuries.

## Conclusion

In conclusion, preparedness for challenging airway management and surgical airway is critical even in patients with isolated facial fractures. The presence of TBI did not predict the need or site of early airway management in facial fracture patients. Facial fracture patients comprise a unique group when evaluating the need for early airway management.

## Data Availability

The datasets generated and/or analysed during the current study are not publicly available due upcoming analyses concerning other study setups but are available from the corresponding author on reasonable request.

## Abbreviations

GCS: Glasgow Coma Scale
ONP: oro-naso-pharyngeal
TBI: Traumatic brain injury
ZMO: zygomatic-maxillary-orbital
